# The E592K variant of SF3B1 creates unique RNA missplicing and associates with high-risk MDS without ring sideroblasts

**DOI:** 10.21203/rs.3.rs-2802265/v1

**Published:** 2023-04-14

**Authors:** In Young Choi, Jonathan Ling, Jian Zhang, Eric Helmenstine, Wencke Walter, Riley Bergman, Céline Philippe, James Manley, Kevin Rouault-Pierre, Bing Li, Daniel Wiseman, Madhu Ouseph, Elsa Bernard, Xiao Li, Torsten Haferlach, Salman Fazal, Tania Jain, Christopher Gocke, Amy DeZern, W. Brian Dalton

**Affiliations:** Johns Hopkins University School of Medicine; Johns Hopkins University; Columbia University; Johns Hopkins University; MLL Münchner Leukämielabor GmbH; Vanderbilt University; Barts Cancer Institute, Queen Mary University of London; Columbia University; Barts Cancer Institute - Queen Mary University of London; Institute of Hematology and Blood Diseases Hospital, Chinese Academy of Medical Sciences and Peking Union Medical College; The University of Manchester; Weill Cornell Medical College; Memorial Sloan Kettering Cancer Center; Shanghai Jiao Tong University Affiliated Sixth People’s Hospital; MLL Munich Leukemia Laboratory; Allegheny Health Network; Sidney Kimmel Comprehensive Cancer Center at Johns Hopkins University; Johns Hopkins University School of Medicine; Johns Hopkins University School of Medicine; Johns Hopkins Medicine

## Abstract

Among the most common genetic alterations in the myelodysplastic syndromes (MDS) are mutations in the spliceosome gene *SF3B1*. Such mutations induce specific RNA missplicing events, directly promote ring sideroblast (RS) formation, generally associate with more favorable prognosis, and serve as a predictive biomarker of response to luspatercept. However, not all *SF3B1* mutations are the same, and here we report that the E592K variant of *SF3B1* associates with high-risk disease features in MDS, including a lack of RS, increased myeloblasts, a distinct co-mutation pattern, and decreased survival. Moreover, in contrast to canonical SF3B1 mutations, E592K induces a unique RNA missplicing pattern, retains an interaction with the splicing factor *SUGP1*, and preserves normal RNA splicing of the sideroblastic anemia genes *TMEM14C* and ABCB7. These data expand our knowledge of the functional diversity of spliceosome mutations, and they suggest that patients with E592K should be approached differently from low-risk, luspatercept-responsive MDS patients with ring sideroblasts and canonical SF3B1 mutations.

## Introduction

*SF3B1* is the most mutated spliceosome gene in MDS, with a frequency of > 30%^1^. The mutations are primarily missense substitutions that induce neomorphic RNA missplicing in thousands of junctions, which in turn alter expression of hundreds of genes in diverse pathways^2^. This missplicing has been implicated in many MDS phenotypes, including dysfunctional iron metabolism, formation of ring sideroblasts, activation of innate immune signaling, and promotion of hematopoietic stem cell self-renewal^3–8^. *SF3B1* mutations also contain prognostic value in MDS, associating with more indolent disease, though with important exceptions^9–12^. In MDS treatment, *SF3B1* mutations are among the criteria used to determine eligibility for luspatercept, and they are the direct or indirect targets of investigational therapies^13^. Thus, *SF3B1* mutations figure prominently in the research of—and clinical practice for—many MDS patients.

Not well understood is whether—and how—distinct *SF3B1* mutation hotspots differentially affect disease features and/or the RNA missplicing events that drive them. Previously, in an analysis of patients and cell models with *SF3B1* exon 14–15 mutations, we found that the K666N variant was enriched in high-risk MDS and produced an asymmetrical lack of missplicing events that are induced by K700E and H662Q mutations^14^. Here we report the results of extending this approach to a larger cohort of patients that included exon 13–16 mutations and additional cell models. This analysis revealed a striking distinctiveness in the *SF3B1* mutation E592K, which has implications for the understanding and management of *SF3B1*-mutant MDS.

## Materials/subjects And Methods

### Patients

Mutation-agnostic acquisition of *SF3B1* mutations from MDS and AML cases and associated clinical parameters came from the Johns Hopkins Sidney Kimmel Cancer Center, the Vanderbilt-Ingram Cancer Center, the Chinese Academy of Medical Sciences, the Munich Leukemia Laboratory, the Allegheny Health Network Cancer Institute, Project Genie (http://genie.cbioportal.org/), and manual extraction from 82 published studies (supplemental References)^15^. Additional E592K cases and their clinical parameters were deliberately obtained from The University of Manchester, Tong University Affi liated Sixth People’s Hospital, Weill Cornell Medicine, and Memorial Sloan Kettering Cancer Center. Because breadth of gene panels varied among patients, co-mutation analysis included those cases in which at least a set of 35 genes were sequenced, representing a compromise between maximum sample inclusion and maximum gene inclusion. For EZH2 co-mutation analysis, cBioportal Oncoprinter (https://www.cbioportal.org/oncoprinter) was applied to all MDS cases from the MSK Myelodysplastic, Project GENIE, and IPSS-M cohorts^10,15,16^. For leukemia-free survival of E592K patients, the IPSS-M (https://mds-risk-model.com) and SEX-GSS (https://mds.itb.cnr.it/#/mds/home) calculators were used^10,11^. Use of deidentified patient data were approved by the Institutional Review Boards at the respective institutions.

### Cells

HEK293T, TF1, and K562 cells were obtained from the ATCC. HEK293T cells were grown in DMEM/10% FBS, K562 cells were grown in RPMI/20% FBS, and TF1 cells were grown in RPMI/20% FBS with 2 ng/mL GM-CSF. STR cell line authentication and mycoplasma testing were done upon receipt and routinely thereafter, with last testing done 2/2022.

### Vectors

WT and K700E FLAG-*SF3B1* sequences were subcloned from Addgene plasmids 82576 and 82577 into pDONR-A-HYG (Addgene 29635) to make pENTR-*SF3B1*-WT and pENTR-*SF3B1*-K700E. Site-directed mutagenesis with overlap extension PCR then created pENTR plasmids for the E592K, E622D, K666R, K666N, R625H, K741N, and E902K variants of *SF3B1*, and these were subcloned into lentiviral vector pLX301 (Addgene 25895). Plasmids used for *SF3B1* affi nity purification have been previously described^17^. Site-directed mutagenesis of the p3xFLAG-CMV-14-His6-FLAG-SF3B1 vector was done by overlap extension PCR to make the E592K vector.

### Transcriptome analysis

For HEK293T cells, pLX301 plasmids were transfected using Lipofectamine 3000, 24h later cells were selected with puromycin for 48h, puro was washed out for 24h, and cells were harvested. For stable transduction, pLX301 lentivirus was produced as previously described^18^. TF1 cells were transduced and four independent clones per genotype were puro selected from single cells. For K562 cells, duplicate independent polyclonal populations per genotype were puro selected. RNA isolation, cDNA synthesis, endpoint PCR, and quantitative PCR were performed as described^18^. Primer sequences are in Supplementary Table 1. RNA-seq libraries from TF1 clones were constructed using TruSeq Stranded Total RNA Library Prep. Sequencing was performed on a NovaSeq S1 flowcell. Reads were aligned using STAR^19^. Splicing analysis was performed using ASCOT and gene expression using featureCounts^20,21^. Percent spliced in (PSI) values for junctions were determined by dividing inclusion split-read counts by the total split-read counts at the corresponding constitutive donor or acceptor sites, using a minimum coverage of 15 split-reads per junction. Junctions of interest were visualized from RNA-seq reads from primary MLL samples with the UCSC genome browser for 2 E592K, 10 E622D, 6 K666N, 9 K666R, 12 K700E, and 12 WT samples, using the ADD function (combining reads and normalizing track height for samples in each mutation group). Junction validation with endpoint PCR was done on independent bone marrow CD34 + MDS samples at Barts Cancer Institute.

### Affinity of Purification *SF3B1*-Associated Proteins

A small-scale protocol was applied to both HEK293T and TF1 cells as previously described, except that for TF1, 10 million cells were used and proteins were eluted with 30 μL (5 μg/μL) 3X FLAG peptide because cells had only one affi nity tag (FLAG) attached to SF3B1^17^.

### Western Blotting

For immunoblotting of *SF3B1*/FLAG-*SF3B1* proteins in transiently-transfected HEK293T and stably-transduced TF1 and K562 cells in which transcriptome analysis was done in parallel, Western blotting using a mouse anti-human-*SF3B1* antibody (Abcam #172634) at 1:1000 dilution was used. Immunoblotting following affi nity purification of SF3B1 in HEK293T and TF1 cells was performed as previously described, and primary antibodies were: anti-SF3B1 (Bethyl Laboratories, A300–996A, 1:1,000), anti-ACTIN (Sigma, A2066, 1:2,000), anti-DYKDDDDK (GenScript, A00187, 1:1,000), anti-*SUGP1* (Bethyl Laboratories A304–675A-M, 1:1,000), and anti-PHF5A (Proteintech 15554–1-AP, 1:1000)^17^. Secondary antibodies were: Donkey anti-Rabbit IgG (LI-COR, 926–68073, 1:5,000) and Goat anti-Mouse IgG (LI-COR, 926–32210, 1:5,000).

## Results

**Figure 1:**
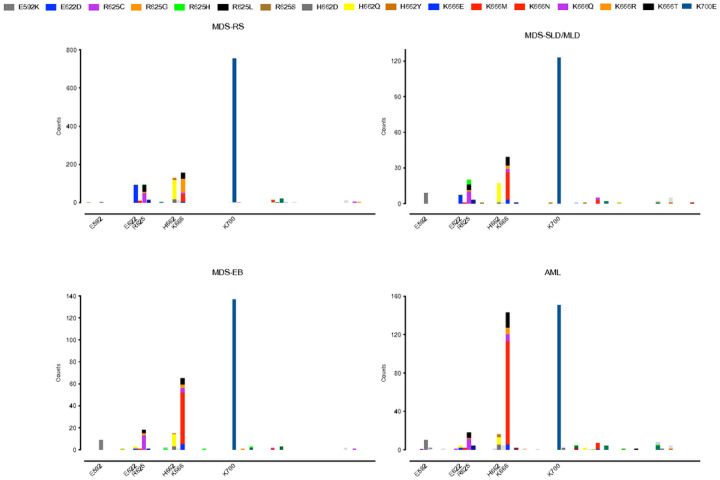
Distribution of exon 13–16 SF3B1 mutations within WHO 2016 classifications of MDS and AML. Amino acid positions are shown along the x axis, and individual variants are counted along the y axis according to the legend above the graphs. E592K and K666N are increased, while E622D and K666R are decreased, in higher-risk disease types. RS = ring sideroblasts. SLD/MLD = single-lineage dysplasia/multi-lineage dysplasia. EB = excess blasts.

Combining patient data from five institutions, publicly available databases, and published literature, we established a dataset of 2,288 patients with *SF3B1*-mutant MDS or AML in which exons 13 through 16 had been sequenced. We first determined how *SF3B1* mutations partitioned into WHO 2016 classifications, as these data were available for virtually all patients. This distribution showed several asymmetries (Fig. 1 and Supplementary Fig. 1). Consistent with our previous report^14^, K666N was enriched in higher-risk disease types: only 2.1% (28/1327) in MDS-RS vs 8.7% (21/242) in MDS-SLD/MLD, 17.3% (46/266) in MDS-EB, and 25.8% (103/399) in AML (p < 0.0001 for all). With the inclusion of cases in which exon 13 had been sequenced, the distribution also revealed enrichment of a variant in this exon, E592K, in higher-risk disease: only 0.08% (1/1327) in MDS-RS vs 3.7% in (9/242) in MDS-SLD/MLD, 3.4% (9/266) in MDS-EB, and 2.5% (10/399) in AML (p < 0.0001 for all). Conversely, E622D was decreased in higher-risk disease: 6.9% (92/1327) in MDS-RS vs 2.9% (7/242) in MDS-SLD/MLD (p < 0.05), 0.4% (1/266) in MDS-EB (p < 0.0001), and 0.5% (2/399) in AML (p < 0.0001). K666R also decreased: 5.4% (72/1327) in MDS-RS vs 1.2% (3/242) in MDS-SLD/MLD, 1.1% (3/266) in MDS-EB, and 1.8% (7/399) in AML (p < 0.01 for all). These data confirm and extend the scope of asymmetrical partitioning of distinct *SF3B1* hotspots in low- and high-risk disease.

**Figure 2:**
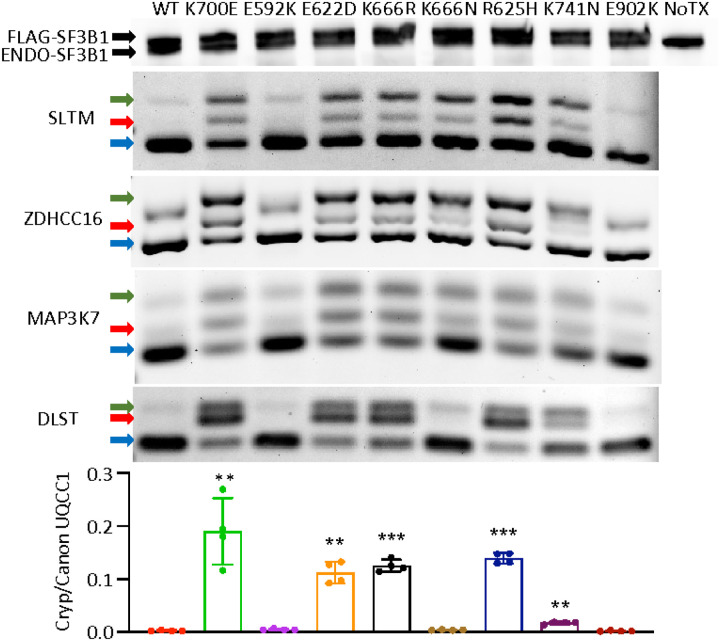
Asymmetric RNA missplicing by distinct SF3B1 mutation hotspots. HEK293T cells were transfected with constructs expressing FLAG-SF3B1 variants. Top row is Western blotting with anti-SF3B1 antibody, showing FLAG-SF3B1 and endogenous SF3B1 at similar levels. Endpoint PCR used isoform-competitive primers, with arrows for canonical (blue), cryptic (red), and heteroduplex (green) forms. Cryptic vs canonical UQCC1 was quantified as a ratio between two separate isoform-specific qPCRs.

We next examined RNA splicing events produced by those *SF3B1* mutations with the most significant asymmetric partitioning. We expressed FLAG-tagged codon-optimized constructs with the E592K, E622D, K666N, and K666R mutations in HEK293 cells, along with wild type *SF3B1* and the dominant K700E mutation. For additional comparison, we expressed variants from solid tumors that are rare (R625H, K741N) or absent (E902K) in myeloid malignancies^22^. These transfections produced a comparable level of endogenous and exogenous *SF3B1* protein (Fig. 2). Upon expression of most hotspots, we observed missplicing in junctions known to be affected by *SF3B1* mutations, including *SLTM*, *ZDHHC16* and *MAP3K7* (Fig. 2)^2,6^. This included MDS mutations K700E, E622D, K666R, and K666N but also solid tumor hotspots R625H and K741N, the last of which produced lower magnitude missplicing as recently reported in the context of uveal melanoma^23^. In contrast, these junctions were not misspliced by E902K, which showed its own unique missplicing pattern as was noted in TCGA E902K bladder cancer samples (Supplementary Fig. 2)^22^. We also found that two other mutant *SF3B1*-associated junctions, in *DLST* and *UQCC1*, were misspliced to high magnitude by most mutations but were unaffected by K666N, confirming that the pattern of missplicing by K666N is different from that of other MDS/AML-associated hotspots^14,24^. Notably, this pattern occurred with K666N, but not K666R, demonstrating these variants are not functionally equivalent even though they affect the same starting amino acid, a phenomenon also observed with variants in the yeast homologue of *SF3B1*^25^. Finally, conspicuously absent was any missplicing of these events by E592K. Coupled with its enrichment in high-risk disease, this distinct missplicing pattern motivated us to investigate E592K further.

**Figure 3:**
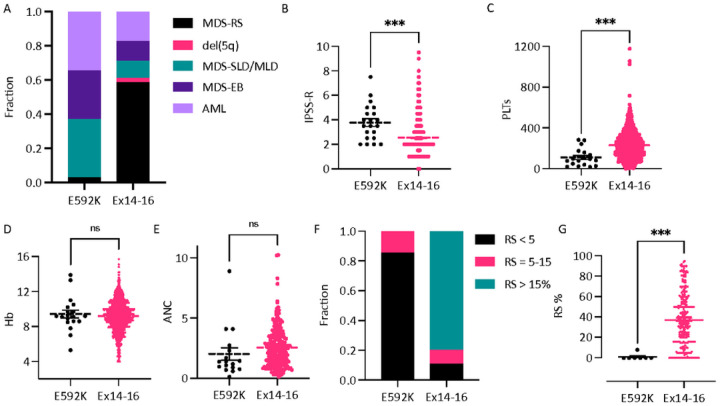
Clinical parameters of MDS patients with the E592K variant of SF3B1. Compared to cases with exon 14–16 mutations, patients with E592K have A) higher risk WHO 2016 classifications, B) higher IPSS-R, C) lower platelets, D-E) similar hemoglobin (Hb) and absolute neutrophil count (ANC), and E-F) nearly-absent ring sideroblasts (RS).

**Figure 4:**
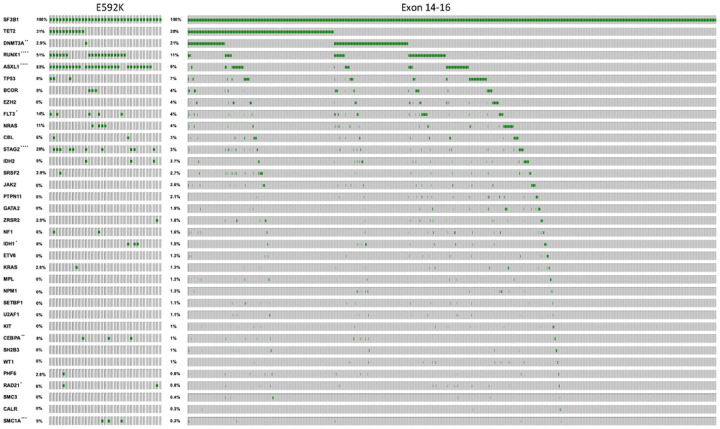
Co-mutation landscape of SF3B1-mutant MDS and AML. All E592K-mutated cases are shown on the left, and all exon 14–16-mutated cases on the right. *p<0.05, **p<0.01, ***p<0.001, ****p<0.0001.

**Figure 5:**
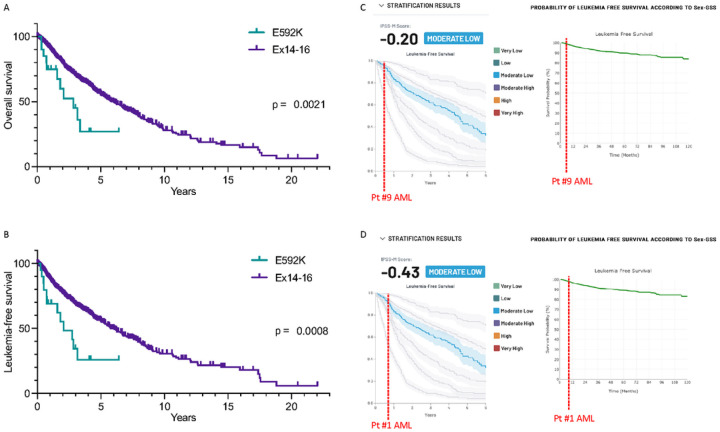
MDS patients with E592K have poor survival. A) Overall and B) leukemia-free survival of all SF3B1-mutant MDS patients. p-values are for Log-rank (Mantel-Cox) tests. C) Example of understaging of Pt #9, who developed AML 6 months after diagnosis but is stratified as moderate low risk with median LFS of 4.5 years by the IPSS-M (left) and a median LFS of >10 years by Sex-GSS (right). D) Understaging of Pt #1, who developed AML 9 months after diagnosis.

We characterized the clinical features of E592K patients in more detail. The hotspot-agnostic collection of all patients with exon 13–16 sequencing had produced 29 cases of E592K. By specifically seeking out additional cases from multiple institutions, we gathered a total of 39 patients with E592K-mutated myeloid neoplasms, 35 of which were MDS or AML (Supplementary Table 2). This expanded E592K cohort also showed enrichment in higher-risk 2016 WHO classifications, and they had higher IPSS-R scores and lower platelets than cases with exon 14–16 mutations (Fig. 3A–C). Hemoglobin and WBC were not significantly different (Fig. 3D–E). E592K cases also had a notable lack of RS (Fig. 3F), with only one instance of low-blast E592K MDS reporting any RS, at 8%, therein being the only E592K patient to meet WHO 2016 criteria for MDS-RS (> 5% RS if *SF3B1* mutation present). By contrast, for low-blast MDS with exon 14–16 mutations in which exact RS percentages were available, the average was 37%, consistent with the known pathological and mechanistic links between mutant *SF3B1* and RS in MDS (Fig. 3G)^5,26^. E592K MDS/AML also had a different co-mutation profile (Fig. 4 and Supplementary Fig. 3–5). A striking distinction was the nearly ubiquitous co-mutation of ASXL1 (83%) in E592K cases, compared to only 9% in exon 14–16 disease (p < 0.0001). High ASXL1 co-mutation characterized both low and high blast E592K cases, suggesting this relationship occurs early in disease evolution (Supplementary Fig. 3–5). Also notable was the near absence of DNMT3A co-mutations (2.9%) with E592K, despite DNMT3A being the 2nd most commonly co-mutated gene (21%) in exon 14–16 disease (p < 0.01). In fact, the one DNMT3A mutation in E592K MDS was a VAF of 2.3% in a sample in which the VAFs for E592K and ASXL1 mutations were 20%, raising the possibility that the DNMT3A mutation was in a separate clone with wild type *SF3B1*. Furthermore, E592K patients had increased co-mutations of RUNX1 (51% vs 11%) and STAG2 (29% vs 3%) (p < 0.0001 for both). Finally, consistent with these high-risk MDS clinical features, both overall survival and leukemia-free survival were markedly shorter in E592K patients (Fig. 5A–B), including two patients (#9 and #1) who progressed from MDS to AML at 6 and 9 months, respectively, despite lower-risk prognostication scores from both the IPSS-M and SEX-GSS (Fig. 5C–D)^10,11^.

**Figure 6:**
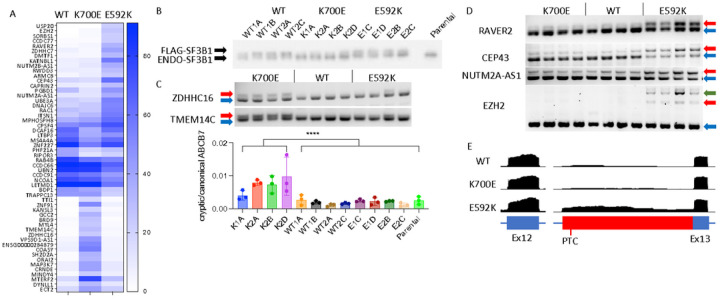
E592K induces unique RNA missplicing events. TF1 cells transduced with different SF3B1 variants were analyzed by RNA-seq, with A) highest-scoring ΔPSIs shown. B) Western blot with anti-SF3B1 antibody. C) Endpoint PCR/qPCR validation of K700E-specific missplicing events. D) Validation of E592K-specific events. E) RNA-seq reads from TF1 cells showing the cryptic event in EZH2. PTC = premature termination codon.

Because our initial splicing analysis merely showed the absence of known *SF3B1*-mutant events in E592K cells, we next sought missplicing events specifically induced by this variant. To do so, we stably expressed the WT, K700E, and E592K mutations through lentiviral delivery in TF1 cells, isolated multiple independent clones for each genotype, and performed RNA-seq to quantify percent spliced in (PSI) values for all splice junctions (Fig. 6A). K700E produced a characteristic pattern of increased expression of junctions using alternative 3’ acceptors, with high magnitude missplicing of genes like *MAP3K7* and *ZDHCC16*, as expected. In contrast, the E592K mutation produced a fundamentally different pattern of missplicing, with its affected genes nonoverlapping with those misspliced by K700E, and vice-versa. Western blotting showed that exogenous FLAG-tagged *SF3B1* was equal or less than the endogenous *SF3B1* form, not overexpressed above it (Fig. 6B). We then validated several missplicing events with endpoint and quantitative PCR assays. For K700E-specific junctions, this included missplicing of *ZDHHC16*, *TMEM14C*, and *ABCB7* (Fig. 6B–C). For E592K-specific junctions, this included *RAVER2*, *CEP43*, *NUTM2A-AS1*, and *EZH2* (Fig. 6D). Interestingly, the EZH2 missplicing event was an alternate acceptor in intron 12, creating a premature termination codon predicted to activate nonsense-mediated decay (Fig. 6E). We further validated the hotspot specificity of these events in two other cell contexts: transiently-transfected HEK293T and stably-transduced K562 cells (Supplementary Fig. 6). In all cases, K700E-dependent and E592K-dependent missplicing events were present and distinct. Of note, missplicing of *TMEM14C* and *ABCB7* were recently shown to drive ring sideroblast formation in iPSCs derived from *SF3B1*-mutant MDS^5^. Both genes were clearly misspliced by K700E, but not by E592K, consistent with the lack of sideroblastic anemia in E592K patients. Together, these data show that the E592K variant of *SF3B1* has a unique pattern of RNA missplicing.

**Figure 7:**
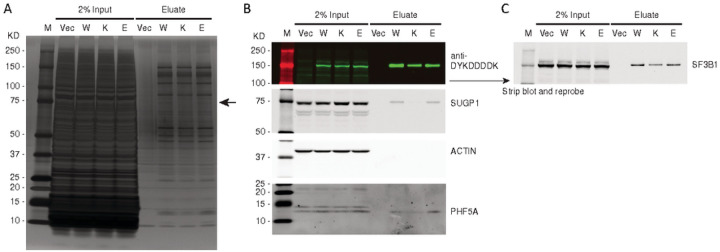
The E592K variant preserves association of SF3B1 with SUGP1. HEK293T cells were transfected with His6-FLAG-SF3B1 variants and subjected to affi nity purification with anti-DYKDDDDK (FLAG) antibody. A) Silver-stained protein gel, with arrow pointing to the size of SUGP1, which is decreased in K700E but not E592K eluate. B) Western blot showing decreased SUGP1 in K700E, but not E592K, eluate. PHF5A is present with all SF3B1 variants. C) Reprobing with anti-SF3B1 shows native and His6-FLAG-tagged protein levels.

In addition to shared RNA missplicing events, the most well-studied *SF3B1* hotspot mutations also share a specific biochemical defect: disruption of the interaction between *SF3B1* and *SUGP1*^17^. This disruption is not incidental to missplicing but directly mediates it; inactivation of *SUGP1* recapitulates *SF3B1*-mutant missplicing and overexpression of *SUGP1* partially rescues it^17^. We therefore asked whether E592K, with its nonoverlapping missplicing events, might preserve the interaction of *SF3B1* with *SUGP1*. Indeed, when His6-FLAG-*SF3B1* variants were introduced into HEK293T cells and affi nity purified using anti-DYKDDDDK (FLAG) antibody and cobalt beads, the association of His6-FLAG-*SF3B1* with endogenous *SUGP1* was disrupted by K700E but not by wild type or E592K *SF3B1* (Fig. 7). We then asked whether E592K might instead disrupt the interaction between *SF3B1* and *PHF5A*, given that E592 is at the interface with *PHF5A*^27^. However, this interaction was preserved by each His6-FLAG-SF3B1 variant (Fig. 7). We also observed these effects in a second cell context, TF1 cells expressing FLAG-*SF3B1* variants (Supplementary Fig. 7). Together, these data indicate that, consistent with its induction of unique RNA missplicing events, the E592K variant does not participate in the disruption of the *SF3B1*-*SUGP1* interaction that drives the cryptic splicing of other *SF3B1* mutations.

**Figure 8:**
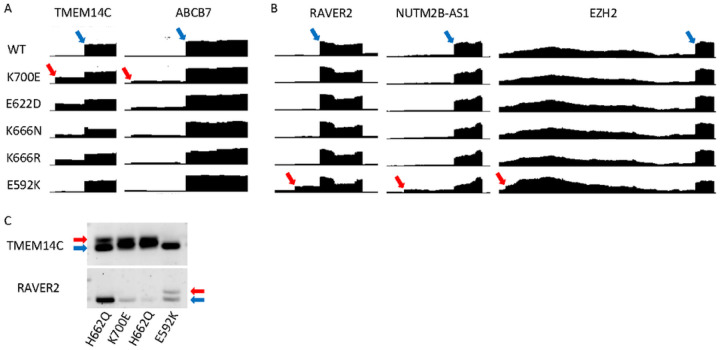
E592K exhibits unique RNA missplicing in primary MDS samples. RNA-seq read distribution in the MLL cohort shows that E592K exhibits A) canonical TMEM14C and ABCB7 missplicing, and B) cryptic RAVER2, NUTM2B-AS1, and EZH2 missplicing. C) Distinct TMEM14C and RAVER2 missplicing was validated in marrow CD34+ cells from an independent patient by endpoint PCR.

Finally, we identified and analyzed RNA-seq from two patients with E592K mutation, compared to other *SF3B1* mutations, from the recent MLL cohort of spliceosome-mutant myeloid malignancy patients^28^. Inspection of the junctions validated in our cell models also demonstrated the same specificity of missplicing in these primary patient samples, when compared to other *SF3B1* mutations (Fig. 8A). In addition, endpoint PCR validation of TMEM14C and RAVER2 from a third primary E592K sample at a separate institution demonstrated the same hotspot specificity of RNA missplicing (Fig. 8B), validating the findings from our cell models.

## Discussion

Here we show the E592K variant of *SF3B1* produces unique RNA missplicing and associates with high-risk MDS. These results have several implications. First, the distinctiveness of E592K informs the pathobiology of *SF3B1*-mutant MDS. Clough et al elegantly showed that RS are formed by TMEM14C and ABCB7 missplicing in MDS-derived iPSCs with the G742D variant of *SF3B1*^5^. These missplicing events have been seen with other hotspots, including K700E, which we corroborated here^2,29^. Cells with E592K, on the other hand, preserved canonical splicing of these genes, and cases of E592K MDS lacked RS. Other events that have been implicated in *SF3B1*-mutant MDS pathobiology include innate immune activation by missplicing of MAP3K7 and IRAK4, enhanced self-renewal by MECOM missplicing, impaired erythropoiesis by COASY missplicing, and hepcidin suppression by ERFE missplicing; all these genes were also canonically spliced in E592K cells (Supplementary Fig. 7)^3,4, 6–8^. A distinct E592K pathobiology is also suggested by its high co-occurrence with ASXL1/RUNX1/STAG2 mutations and mutual exclusivity with DNMT3A mutations, a starkly different pattern than that of other *SF3B1* hotspots. As with most co-mutation patterns in cancer, it is unclear whether these co-occurrences are driven by synergism or permissiveness—and whether the mutual exclusivity is driven by antagonism or redundancy. Interestingly, the same pattern of co-occurrence (ASXL1/RUNX1/STAG2) and mutual exclusivity (DNMT3A) occurs with loss-of-function EZH2 mutations in MDS (Supplementary Fig. 8)^10,15^. It is therefore intriguing that E592K missplicing produced a frameshifted EZH2 transcript in our studies here, and it is tempting to speculate this may create ‘mutant EZH2-ness’ in E592K cells, an area for future investigation. If so, mutual exclusivity due to redundancy might be expected between mutant EZH2 and E592K. While there were indeed no EZH2 mutations in the 39 E592K patients here, the low overall frequency (4%) of EZH2 in *SF3B1*-mutant myeloid malignancies means a larger cohort would be needed for significance testing of this particular pair. Nonetheless, the distinctiveness of E592K here shows that certain MDS phenotypes associated with *SF3B1* mutation, including sideroblastic anemia, are not inevitable pathobiological outcomes of RNA missplicing by all *SF3B1* variants.

Second, there are implications for MDS classification. Recently, both the International Consensus Classification (ICC) and the 5th edition of the World Health Organization (WHO) classification of myeloid neoplasms created nosologic entities for *SF3B1*-mutant MDS^30,31^. These are based on the International Working Group (IWG) findings that *SF3B1* mutation, low blasts, and lack of certain co-occurring genetic aberrations defined an indolent MDS with a shared pathobiology of idiosyncratic RNA missplicing that was more homogeneous than the MDS-RS classification^32^. This was due in part to exclusion of *SF3B1*-unmutated MDS-RS which had greater myeloid/megakaryocyte dysplasia, more TP53 co-mutations, and poorer outcomes. Both the ICC and WHO criteria require *SF3B1* mutation, low blasts, cytopenias, dysplasia, and lack of multi-hit TP53/del(5q)/−7/complex cytogenetics; the ICC also requires *SF3B1* VAF > 10% and lack of RUNX1/del(7q)/abn3q26.2; and the WHO allows for > 15% RS to substitute for *SF3B1* mutation. As we have seen here, E592K patients do not fit these groups: they have unique RNA missplicing, higher blasts, lack of RS, increased RUNX1 mutations, and poorer prognosis. These differences make an argument for excluding those E592K cases that would otherwise meet criteria for these entities. They also emphasize the need to consider specific hotspot mutations in future classification efforts. This should be the precise mutation, not just the affected amino acid, as we demonstrated different patterns of disease partitioning and missplicing between K666R and K666N.

Third, our data have implications for MDS prognostication. Along with low blasts, shallow cytopenias, and certain cytogenetic abnormalities such as del(11q), *SF3B1* mutations have been consistently associated with better MDS outcomes in multiple independent datasets^1, 9–12^. Accordingly, new MDS prognosis scoring tools incorporating mutational data have generally weighted *SF3B1* mutations favorably, although there is important context-dependence that overrides this favorability, such as increased blasts and aberrations such as del(5q), RUNX1 mutation, and others^10–12^. However, given the much larger patient cohorts that would be required, prognostication tools do not yet separately weight the myriad individual variants of mutated genes (*SF3B1* or otherwise) into their scores. By the same token, a comprehensive multivariate analysis of the prognosis of E592K MDS would require a larger patient cohort than ours here. Nonetheless, while increased blasts or co-occurring RUNX1 mutations would lead to high-risk scores for many E592K patients, others would be understaged by these tools, as we observed here for two patients with rapid progression to AML (Fig. 5C–D). Thus, our data suggest caution in regarding any E592K patients as low risk. They also highlight the potential added value of incorporating specific hotspot mutations in the eventual next generation of prognostication tools.

Fourth, these results have treatment implications. Luspatercept is indicated for treatment of anemia in ESA-refractory MDS-RS patients based on the phase III MEDALIST trial, which was conducted in this population due to strong associations of MDS-RS and *SF3B1* mutation with response in the phase II PACE-MDS study^33,34^. However, response of MDS-RS patients was similar regardless of mutant *SF3B1* allelic burden in MEDALIST, as well as in a recent real-world cohort^33,35^. These data would suggest that it is sideroblastic anemia (or the late-to-early erythroid progenitor ratio common in MDS-RS, according to long-term PACE-MDS follow up) that is predictive of luspatercept response, more so than *SF3B1* mutation^36^. In the WHO 5th edition, MDS-RS has become MDS-*SF3B1* and does not require RS if there is *SF3B1* mutation, low blasts, and lack of high-risk cytogenetics^31^. Luspatercept would therefore be approved for many low-blast E592K patients, despite the sharp distinctions from MDS-RS patients that we have drawn here. Our data thus also recommend caution in approaching E592K patients like MDS-RS patients that are likely to respond to luspatercept. There are also implications for investigational therapies. Gene therapies that leverage missplicing in spliceosome-mutant cells are promising, but such vectors would need to be not only gene-specific (i.e. *SF3B1* vs *SRSF2*) but, in the case of E592K, hotspot-specific due to its nonoverlapping missplicing^37,38^. Similarly, antisense oligonucleotide therapy aimed at rescuing *BRD9* missplicing showed activity against *SF3B1*-mutant tumors in vivo, but such a therapy would not apply to E592K, which does not missplice *BRD9* (Supplementary Fig. 7)^39^.

Finally, these findings add to our understanding of the functional diversity of spliceosome mutations. After these mutations were first discovered as conspicuously enriched in myeloid neoplasms in a mutually exclusive manner, efforts have sought unifying downstream functional effects that might explain this occurrence pattern^40^. This has revealed shared phenotypes, such as convergent disruption of certain pathways, creation of genotoxic R-loops, and sensitization to further disruption of the spliceosome^6, 41–44^. At the same time, bulk and then single-cell analyses have shown that spliceosome mutations are not always mutually exclusive, with multiple mutations sometimes selected for in the same cells^24^. This indicates that different spliceosome mutations can confer different, even complementary, advantages to cancer cells, and the mutual exclusivity that does occur may be more from the splicing toxicity of combining certain mutations than from functional redundancy^24^. Consistent with this, studies have shown direct functional differences between spliceosome mutations: the missplicing events induced by mutations in different spliceosome genes (i.e. *SRSF2* vs *U2AF1*, etc) are nonoverlapping, the two dominant hotspot mutations in *U2AF1* missplice different genes, and some less common *SRSF2* and *U2AF1* variants only partially or “dually” recapitulate the RNA missplicing of hotspot mutations^2, 45–48^. For *SF3B1* mutations, their asymmetric partitioning among cancer types (i.e. K700E/H662Q in MDS, R625C/R625H in uveal melanoma, G642D in CLL, etc.) first suggested the mutations may have functional differences^40,49,50^. Later, Seiler et al noted different missplicing events in TCGA RNA-seq from bladder tumors with the E902K variant, events that we experimentally confirmed here^22^. For other *SF3B1* hotspots, studies have described differences of degree in RNA missplicing events, including K700E vs R625C/R625H in primary samples, R625H vs K741Q vs others in isogenic cells, and K666N vs K700E/H662Q in primary samples and isogenic cells^14,23,24,51^. To these we add E592K, whose missplicing events are differences of kind, nonoverlapping with those of other MDS-associated hotspots—and marked by a different underlying biochemistry that preserves the interaction between *SUGP1* and *SF3B1*. While it remains possible that the important underlying oncogenic effects of E592K are phenotypes still shared with other *SF3B1* hotspots, the distinctiveness of E592K suggests to us that different *SF3B1* variants can promote different kinds of leukemia by inducing different RNA missplicing events. Because these differences can impact our understanding and management of patients with myeloid neoplasms, additional studies of differences between other spliceosome variants are warranted.

## Data Availability

Raw FASTQ files are deposited at the NCBI Sequence Read Archive (SRA) under accession number SRP##########.
